# MCMV Centrifugal Enhancement: A New Spin on an Old Topic

**DOI:** 10.3390/pathogens10121577

**Published:** 2021-12-03

**Authors:** Trevor J. Hancock, Morgan Lynn Hetzel, Andrea Ramirez, Tim E. Sparer

**Affiliations:** Department of Microbiology, University of Tennessee, Knoxville, TN 37996, USA; thancoc5@vols.utk.edu (T.J.H.); mhetzel1@vols.utk.edu (M.L.H.); aramir11@vols.utk.edu (A.R.)

**Keywords:** centrifugal enhancement, MCMV, cytomegalovirus, flow virometry, extracellular vesicles

## Abstract

Human cytomegalovirus (HCMV) is a ubiquitous pathogen infecting a majority of people worldwide, with diseases ranging from mild to life-threatening. Its clinical relevance in immunocompromised people and congenital infections have made treatment and vaccine development a top priority. Because of cytomegaloviruses’ species specificity, murine cytomegalovirus (MCMV) models have historically informed and advanced translational CMV therapies. Using the phenomenon of centrifugal enhancement, we explored differences between MCMVs derived in vitro and in vivo. We found centrifugal enhancement on tissue culture-derived virus (TCV) was ~3× greater compared with salivary gland derived virus (SGV). Using novel “flow virometry”, we found that TCV contained a distinct submicron particle composition compared to SGV. Using an inhibitor of exosome production, we show these submicron particles are not extracellular vesicles that contribute to centrifugal enhancement. We examined how these differences in submicron particles potentially contribute to differing centrifugal enhancement phenotypes, as well as broader in vivo vs. in vitro MCMV differences.

## 1. Introduction

Human cytomegalovirus (HCMV) is a highly pervasive β-herpesvirus infecting approximately 40–90% of adults worldwide [1]. HCMV is spread through contact with infected bodily secretions or vertically from infected mothers [1]. Once exposed, typical infection is asymptomatic causing mild disease after which CMV establishes a lifelong infection of their host [1,2]. Primary infection or reactivation of HCMV in an immunocompromised individual (i.e., HIV/AIDS, cancer patients and organ transplant recipients) is associated with significant morbidity and mortality [3,4,5,6]. Additionally, in utero infections can be associated with significant neurological sequelae (i.e., sensorineural hearing loss, mental retardation, and microencephaly) [4,6]. Due to the high prevalence and potential disease in vulnerable populations, identification and development of safe and effective treatments and vaccine targets for HCMV are a high priority.

An important characteristic of the β-herpesvirus family is the strict species-specificity of its members. Murine cytomegalovirus (MCMV) is commonly used as an in vivo model system for studying CMV pathogenesis and dissemination [7]. While MCMV and HCMV diverge genetically, disease characteristics and progression mirror each other, making it a valuable system for studying CMV in vivo [7]. Within the HCMV field, inter-strain differences between lab and clinical isolates have furthered the understanding of genetic changes upon serial passage in vitro [8]. For example, HCMV adaptation to cell culture leads to RL13 mutating immediately upon passage while others like UL128 mutate after a handful of passages [8,9]. As such, there is a heightened focus on ensuring genomic integrity and minimizing passages of clinically derived viruses to generate more “in vivo-like” virus strains. While important in HCMV research, re-derivation of “clinical” strains of MCMV is relatively rare. Many labs still use derivatives of the initial Smith and K181 strains from 1954 and ~1980, respectively [10,11]. Significant divergence may exist between commonly used research strains, even those preserved in bacterial artificial chromosomes and environmental isolates [12]. Despite the potential laboratory adaptation of MCMV, the mouse model of CMV allows for the comparison between in vivo vs. in vitro derived viruses, especially with the ability to easily generate large amounts of in vivo virus. These differences would be difficult to study in HCMV and direct clinical isolates.

Previously, differences between in vivo and in vitro CMVs have been documented. In vivo derived MCMVs are more virulent/lethal to neonates compared with viruses grown in tissue culture [13,14]. Similarly, in vivo derived MCMV is more resistant to entry inhibitors such as heparan sulfate proteoglycan (HSPG) binding peptides and is highly resistant to antibody neutralization. This parallels findings with HCMV where direct clinical isolates are resistant to HSPG-entry inhibitors [15,16]. The viral source was also shown to impact their entry mechanism, modifying cell-surface carbohydrate attachment and cellular tropism [17,18]. These differences may be related to in vivo and in vitro differences in the composition of their virus particle [19]. In vivo derived viruses are more homogenous in size, with cell-culture viruses having both individual virions and multicapsid clusters [19]. These differences in the source for MCMV can affect virulence. Salivary derived virus is attenuated in neonatal mice. However, immunosuppression with cyclophosphamide is capable of restoring virulence [14]. This may be due to reactivation of acute infection or a broadening of SG tropism. For example, immune depletion of CD4+ and CD8+ T cells results in salivary gland infection of both acinar epithelial cells and fibroblasts, potentially altering the virus composition/tropism [20]. Depending on where the virion obtains its membrane can also affect particle composition. MCMV that derives its envelope from the inner nuclear membrane consists of a single capsid per virion, whereas those that derive their envelope from cytoplasmic vacuoles can be either single or multi-capsid virions [21]. Additionally the infected cell type determines the production of single vs. multi-capsid virions. Infection of fibroblasts in vitro or lung fibroblasts in vivo results in the production of single and multicapsid viruses [21,22], while salivary gland acinar cells (epithelial cells) produce single capsid virions in vivo pointing to different maturation processes [19]. How these differences in size and shape affect infection could indicate important limitations of in vitro models and hamper subsequent anti-CMV drug development.

In the late 1960s low speed (<2000× *g*) centrifugation was shown to significantly increase the effective titer of MCMV [23]. This spin enhancement was speculated to be due to a decrease in virus dissociation from the cell surface, thus leading to more efficient attachment to the cell [24]. Observations in other systems such as Toxoplasma and Trachoma showed that the increase in infectious titer was related to their size and the speed at which they were centrifuged [25,26]. This enhancement of infection was termed “spinoculation” or “centrifugal enhancement”. With MCMV, centrifugal enhancement was observed for both viruses derived in vivo and in vitro with similar levels of enhancement reported [23]. Both virus clusters and individual virions were infectious and enhanceable with centrifugation [19]. Enhancement occurs for multiple herpesviruses such as: HCMV, guinea pig CMV, KSHV, etc. and other non-herpesviruses as well (like HIV, hantavirus, etc.) and can occur on multiple cell types [27,28,29,30,31]. Interestingly this enhancement was absent in the similarly-sized alpha-herpesvirus, HSV [23]. For HSV and some other viruses, enhancement was eventually achieved using ultracentrifugation and speeds well in excess of those required for MCMV [29,30,32,33,34,35]. Because CMV infections are enhanced following slow speed centrifugation, this may point to entry differences observed for viruses derived in vivo and in vitro. In order to explore these differences, we carried out a series of experiments with in vitro and in vivo grown MCMVs to measure physical characteristics related to our observed entry differences.

Sedimentation of a particle during centrifugation is related to its size. Based on the ~150–200 nm size of CMV, we would anticipate much greater speeds for enhancement if it were due to sedimentation of the virus [19,36]. One possible explanation of the differences between TCV and SGV stocks is the induction of extracellular vesicles (EVs). EVs are small membranous particles secreted by cells that are capable of transferring proteins and nucleic acids among neighboring cells [37]. Released under homeostatic conditions, EVs are divided into different categories based on their size/biogenesis ranging from small exosomes (<200 nm), microvesicles (~200–500 nm), and ectosomes (≥500 nm) [38,39]. These intercellular shuttles represent an emerging area of research, with their roles in viral infections recently being explored [38,40,41,42,43]. In CMV infections, EVs purified with HCMV were found to contain viral proteins on their surface and interior [37,44,45]. These EVs increase the spread rate of HCMV during focal expansion, potentially due to increased delivery of viral proteins [37]. For HSV, viral-EVs impact infection by enhancing plaque formation, delivery of viral proteins, or manipulation of host immune cells [42,43,46,47]. The size, types, and contents of EVs varies based upon the secreting cell/tissue type [48,49]. In vitro and in vivo tissue/cell types could differ following MCMV infection as well as their secreted EVs. We examined the submicron particle composition of MCMVs from cell culture versus salivary gland and whether these particles could be responsible for the centrifugal enhancement that we observed.

## 2. Results

**Figure 1:**
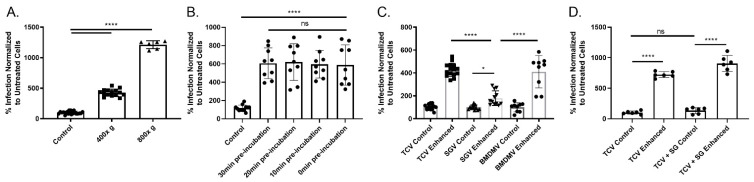
Centrifugal enhancement is predominantly an in vitro phenotype. For all experiments, infection was normalized to a non-centrifuged control. (**A**) Speed determines the level of murine cytomegalovirus (MCMV) enhancement. MCMV adsorbed cells were spun at either 400× or 800× *g* (n ≥ 3). (**B**) Pre-incubation does not affect enhancement. Virus was incubated with cells for the indicated times prior, removed, and centrifuged (n = 3). (**C**) Virus source determines enhancement. Virus derived from tissue culture (TCV), salivary gland (SGV), or bone marrow-derived macrophages (BMDMV) was centrifugally enhanced (400× *g*) (n ≥ 3). (**D**) Salivary gland homogenate does not impact enhancement. Homogenized salivary glands from uninfected mice were mixed with TCV and centrifugally enhanced or not (n = 2). Mean and standard deviation shown for all graphs. For all experiments Tukey’s one-way analysis of variance (ANOVA) with multiple comparisons was performed. Significance: * *p* < 0.05, **** *p* < 0.0001, ns = not significant.

In an effort to better understand centrifugal enhancement, two lower speeds were used to measure enhancement, with significant enhancement of MCMV still occurring (Figure 1A). An ~5-fold increase in plaque formation was observed following a 400× *g* spin and a >15-fold enhancement upon increasing to 800× g spin (*p* < 0.0001) (Figure 1A). Previous studies examined significantly higher centrifugation speeds (1000–2000× *g*’s) and found ~20–50 fold increases in plaque formation demonstrating a direct relationship between centrifugal force and spin enhancement [23]. To investigate the mechanism of centrifugal enhancement, cells were “pretreated” with centrifugation prior to infection. This pretreatment tests whether the act of centrifugation alters the cell’s susceptibility to infection. Enhancement only occurs when virus is present during the centrifugation step [24]. To measure whether adsorption time alters centrifugal enhancement, cells were preincubated with virus for differing times to allow for more adsorption prior to spin enhancement (Figure 1B). Pre-incubation of virus for 10-min intervals up to 30 min prior to centrifugation did not impact enhancement, agreeing with previous findings (Figure 1B) [24,50]. Having established our experimental system, the impact of MCMV source was examined. We suspected potential differences based on our findings that tissue culture, salivary gland, and bone marrow derived macrophage-derived viruses (TCV, SGV, and BMDMV) have subtle differences in their entry process and susceptibility to peptide-based entry inhibitors [15]. Centrifugal enhancement of in vitro-derived viruses (i.e., TCV and BMDMV) had similar increases in infection following enhancement (~5-fold), whereas SGV had only a modest increase in plaque formation (~1.8-fold) (*p* < 0.0001 and *p* < 0.05, respectively) (Figure 1C). To ensure that the SGV prep did not mask the enhancement process, TCV was “spiked” into uninfected salivary glands and its impact on enhancement was assessed (Figure 1D). Despite the addition of salivary gland homogenate to the TCV prep, centrifugal enhancement levels were similar to TCV alone and much greater than the 1.8-fold enhancement observed for SGV alone (*p* < 0.0001). Interestingly, our lab has previously found that BMDMV and TCV had different susceptibilities to anti-CMV peptides pointing to differences in HSPG-dependent entry, with BMDMV conferring an in vivo-like resistance, a phenotype shared by viruses derived from infected mouse salivary gland, spleen, and footpad [15]. If those entry differences correlated with centrifugal enhancement, we would have expected BMDMV to have the lower enhancement seen for SGV.

**Figure 2:**
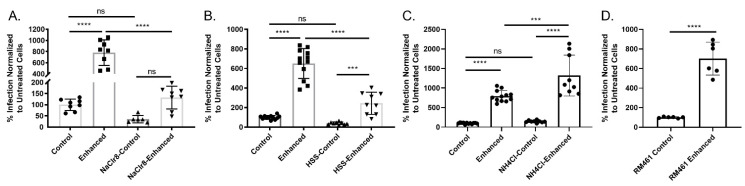
Centrifugal enhancement relies upon heparan sulfate proteoglycans and is MCK2/endocytosis independent. For all experiments, MCMV was either added prior to centrifugation (enhanced) or post-centrifugation (control). (**A**) Enhancement still occurs when heparan sulfate proteoglycan (HSPGs) are reduced with sodium chlorate. Cells were treated overnight with sodium chlorate to reduce sulfation of HSPGs and viral entry (n = 3). (**B**) Enhancement still occurs despite heparin sodium salt (HSS) inhibition. Cells were treated with HSS prior to addition of virus (n = 3). (**C**) Endocytosis does not prevent entry or enhancement in fibroblasts. Cells were treated with ammonium chloride prior to infection (n = 3). (**D**) MCK2 deficient virus still undergoes enhancement. Cell-culture derived RM461 strain of MCMV was used to infect MEF 10.1 fibroblasts (n = 2). In all experiments infection is normalized to untreated, non-enhanced infection. Mean and standard deviation shown for all graphs. For A, B, C Tukey’s one-way ANOVA with multiple comparisons was used. For D, a Student’s one-tailed *t*-test was used for statistical analysis. Significance: *** = *p* < 0.001, **** = *p* < 0.0001, ns = not significant.

As TCV and SGV differed in their susceptibility to HSPG-binding entry inhibitors [15] and there are differences in centrifugal enhancement based on virus source (Figure 1D), one possible explanation of centrifugal enhancement could be the entry process. MCMV entry into fibroblasts is dependent upon host surface HSPGs and occurs through fusion/macropinocytosis at the cell membrane [51,52]. Using TCV due to its unrestricted tropism and large fold change following centrifugal enhancement, cells were treated with sodium chlorate, a chemical inhibitor of heparan sulfate sulfation and centrifugal enhancement measured. Entry was impacted as expected. However, the sodium chlorate treated group still showed an ~5-fold enhancement over their control wells although it was not statistically significant, probably due to the low level of virus infection (Figure 2A). To further demonstrate the importance of HSPGs in the entry and centrifugal enhancement process, cells were pre-incubated with heparin sodium salt, a known blocker of HSPG entry (Figure 2B). As expected, the treated groups had decreased infection but still showed enhancement following centrifugation (*p* < 0.0001). Endocytosis of MCMV occurs in select cell types and is mediated via the viral protein MCK2 [53]. To rule out endocytosis as a mechanism contributing to centrifugal enhancement, ammonium chloride was administered to block the endocytic entry pathway of MCMV [27,51]. Treatment with ammonium chloride did not impact infection in the control group and enhancement still occurred in the centrifuged group (*p* < 0.0001) (Figure 2C). Similarly, an MCK2-deficient strain of MCMV (RM461) [54], which would limit its endocytosis [53], was still enhanced with a low-speed centrifugation (*p* < 0.0001) (Figure 2D).

**Figure 3:**
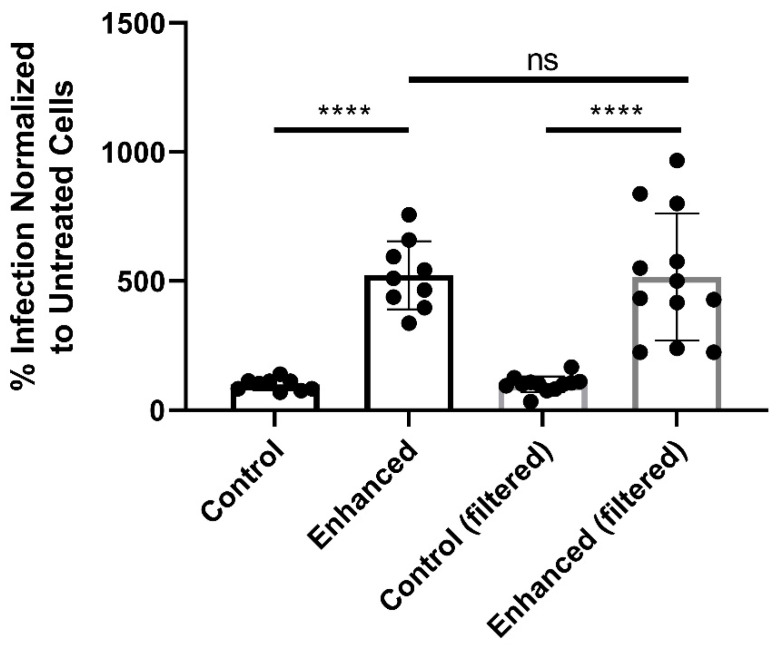
Enhancement is not due to >200 nm particles. 0.2 µm filtration of MCMV does not prevent enhancement. TCV was filtered through a 0.2 µm Millipore filter. Filtered stock was then centrifugally enhanced and normalized to control, non-centrifuged (but still filtered) virus (n ≥ 3). Mean and standard deviation are shown. One-way ANOVA was performed with multiple comparisons. Significance: **** *p* < 0.0001, ns = not significant.

Without a difference in entry mechanisms to explain the differences in enhancement, size could be another possible explanation. Previous MCMV reports noted the presence of >200 nm “multicapsid clusters” containing multiple viral capsids inside a single membrane [19]. These larger particles sedimented with a different density than individual virions, but were similarly infectious [19]. To eliminate these larger clusters, TCV was filtered through a 0.2 µm filter and centrifuged. Despite the absence of particles >200 nm, centrifugal enhancement still occurred when normalized to the non-enhanced filtered control (*p* < 0.0001) (Figure 3). Because enhancement still occurs, the presence/differences in ≤200 nm particles between in vitro and in vivo derived viruses were measured.

**Figure 4:**
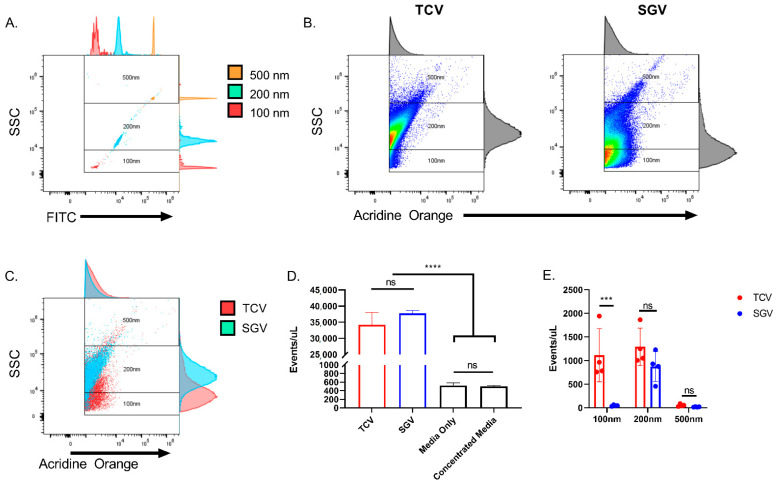
Flow virometry of MCMV identifies distinct TCV vs. SGV particle distributions. (**A**) Sub-micron particle reference standards and gates. 100 nm, 200 nm, and 500 nm beads were run separately and overlaid. (**B**) Flow virometry of tissue culture and salivary gland viruses. Viruses were stained with acridine orange. Nucleic acid positive events were graphed vs. side-scatter and gates corresponding to different sub-micron sizes overlaid. Representative data shown. (**C**) Overlay of TCV and SGV acridine orange-stained viruses. Nucleic acid positive populations in (**B**) were overlaid and distributions compared. For (**A**–**C**) histograms on top and right-hand side show location and distribution of each population. (**D**) Events per microliter of each sample type at equivalent dilutions/viral titers. (**E**) Distribution of nucleic acid-positive events. TCV and SGV nucleic acid positive events are graphed with separate colors representing percentage of events that coincided within each gate (100 nm, 200 nm, and 500 nm). For all, n ≥ 2 and mean +/− standard deviation are shown. For D, one-way ANOVA was performed with multiple comparisons. Two-way ANOVA was performed in (**E**), *** *p* < 0.001, **** *p* < 0.0001, ns = not significant.

To examine the heterogeneity of submicron MCMV particles between TCV and SGV, flow virometry was used to examine virus particle sizes. Thanks to recent advances in flow cytometry detectors and technology, minimum detectable particle size of violet side scatter equipped machines has dropped precipitously [55,56]. Using a Cytek Northern Lights cytometer equipped with a violet (405 nm) laser with violet side scatter set as the trigger, particles as small as 100 nm can be discriminated from noise. Using those settings, particles were gated into 100 nm, 200 nm, and 500 nm approximate size groups (Figure 4A). We utilized the nucleic acid stain acridine orange to stain TCV and SGV derived MCMV and analyzed each via flow cytometry (Figure 4B). Due to the membrane-permeable nature of acridine orange, positive events could constitute enveloped virions, naked capsids, or nucleic acid containing EVs. Virus preparations were first treated with DNase/RNase to remove extracellular DNA/RNA and prevent false staining of damaged particles. In Figure 4B, TCV and SGV plots are shown at equivalent dilutions and titers. When the flow virometry graphs are overlaid, there is a notable difference in their particle size distributions (Figure 4C). TCV has a broader distribution of particle sizes, whereas SGV is predominantly within the 200 nm gate (Figure 4C,E). The event rate for equivalent titered TCV and SGV stocks (~34,000 and 37,000, respectively) was not significantly different at the same dilution (Figure 4D). Additionally, the events detected were significantly greater than the event rate of the FBS-containing media (Media Only ~ 500) or uninfected media concentrated and resuspended as for TCV (Concentrated Media ~ 500) (Figure 4D). When plotted together in Figure 4E, the number of nucleic acid positive events illustrates the differences in sizes of these particles depending on their source. TCV has a nearly 50/50 distribution of particles in the 100 nm and 200 nm gates. Approximately 90% of the SGV stock is within the 200 nm gate with the remainder closely split between 100 nm and 500 nm (Figure 4E). This size distribution of SGV matches electron microscopy observations from the early 1970s stating that SGV has a very homogenous composition [19]. Based on acridine orange staining, TCV and SGV only differ significantly in the 100 nm nucleic acid positive events (*p* < 0.001) (Figure 4E).

**Figure 5:**
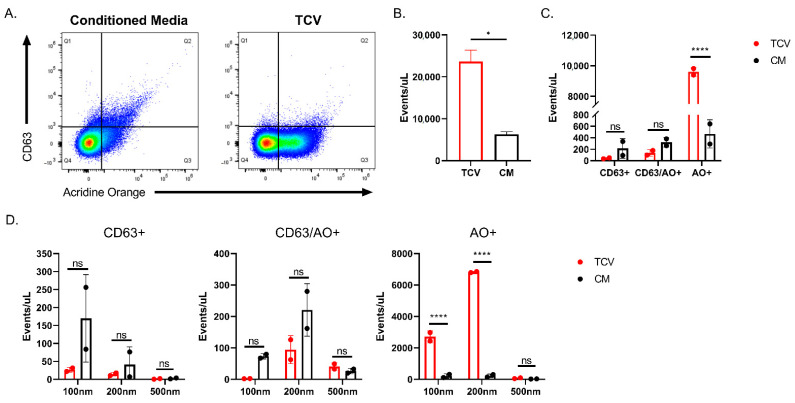
MCMV infection alters MEF 10.1-secreted extracellular vesicles (EVs). (**A**) Comparison of CD63+ and AO+ positive events from uninfected (conditioned media) and infected cells (TCV). Representative FACS plots are shown. (**B**) Event rate of similarly produced conditioned media (CM) and TCV. (**C**) Quantification of events per microliter that were positive in each gate of (**A**). (**D**) Particle size comparison of single and double positive CD63+ and AO+ events for conditioned media and TCV. Representative plots shown. For all, n ≥ 2 and mean +/− standard deviation shown. Student’s two-tailed *t*-test was performed for B. For C and D, two-way ANOVA was performed with multiple comparisons. * *p* < 0.05, **** *p* < 0.0001, ns = not significant.

**Figure 6:**
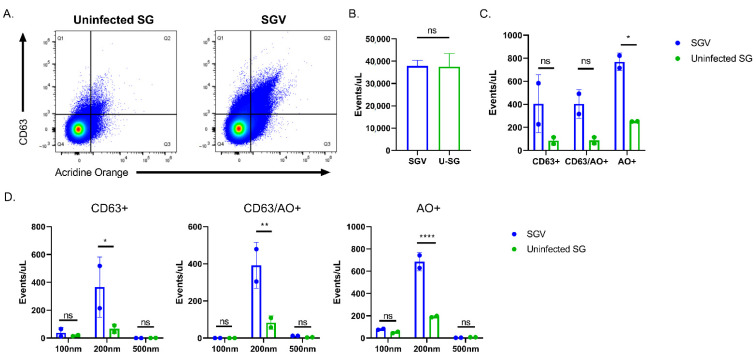
In vivo MCMV infection alters EV composition within the salivary gland. (**A**) Comparison of CD63+ and AO+ positive events for salivary gland homogenate from uninfected (uninfected SG) and infected animals (SGV). Representative FACS plots are shown. (**B**) Event rate of similarly produced uninfected salivary glands (U-SG or Uninfected SG) and SGV. (**C**) Quantification of events per microliter that were positive in each gate of (**A**). (**D**) Particle size comparison of single and double positive CD63 and AO events for uninfected SG and SGV. Representative plots shown. For all, n ≥ 2 and mean +/− standard deviation shown. A Student’s two-tailed *t*-test was performed for (**B**). For (**C**,**D**), two-way ANOVA was performed with multiple comparisons. * *p* < 0.05, ** *p* < 0.01, **** *p* < 0.0001, ns = not significant.

Differences in the production of EVs could explain the differences in particle sizes contained within the TCV and SGV stocks. HCMV infected cells alter their EV production, skewing particle size downwards and increasing their production [37], whereas MCMV’s effect on EV production is unknown. To investigate submicron particle production in vitro and within excised salivary glands, TCV/SGV and supernatants from uninfected cells/tissue were stained with acridine orange and anti-CD63 antibody, which recognizes a Type III lysosomal protein found on EVs [38]. CD63 is also potentially found on HCMV virions due to a similar egress pathway as many EVs [45]. Based on this, CD63+/acridine orange (AO)+ events in ≥200 nm gates could represent either EVs or MCMV. In Figure 5A flow virometry plots show differences in similarly prepared stocks from infected and uninfected fibroblasts (TCV and conditioned media (CM), respectively). As expected, MCMV infection increased total submicron events/µL ~3–4-fold (*p* < 0.05) (Figure 5B). CM and TCV had similar numbers of CD63+ and CD63+/AO+ (Figure 5C). Infection drastically increased the number of AO+ only events (~20-fold) versus CM (*p* < 0.0001) (Figure 5C). Along with having similar numbers of events for CD63+ and CD63+/AO+ groups (Figure 5C), the size of those particles was not significantly different between TCV and CM (Figure 5D). There were trends of decreasing CD63+ and CD63+/AO+ event rates in infected cells, but they failed to reach significance despite CM showing ~2-fold greater events (Figure 5C), driven by 100 nm and 200 nm sizes (Figure 5D). Size distributions of AO+ events shown in Figure 5D demonstrate that TCV produced significantly more particles (both 100 nm and 200 nm sizes (*p* < 0.0001)) than uninfected cells. Most likely these are virus particles. For SGV, equivalent numbers of salivary glands from infected and uninfected mice (SGV and uninfected SG, respectively) were harvested and stained as in Figure 5 (Figure 6A). Similar numbers of total submicron events were detected for both infected and uninfected salivary glands (Figure 6B). Uninfected salivary gland tissue had noticeably fewer CD63+ or CD63+/AO+ events than infected salivary glands (~20% SGV events/µL), but only AO+ single positive events showed statistical significance (*p* < 0.05) with ~3-fold increase when salivary glands were infected (Figure 6C). The increased events in SGV were driven by 200 nm particles in every quadrant (Figure 6D). There was no statistical difference between SGV and uninfected salivary glands positive events in the 100 nm and 500 nm sizes.

**Figure 7:**
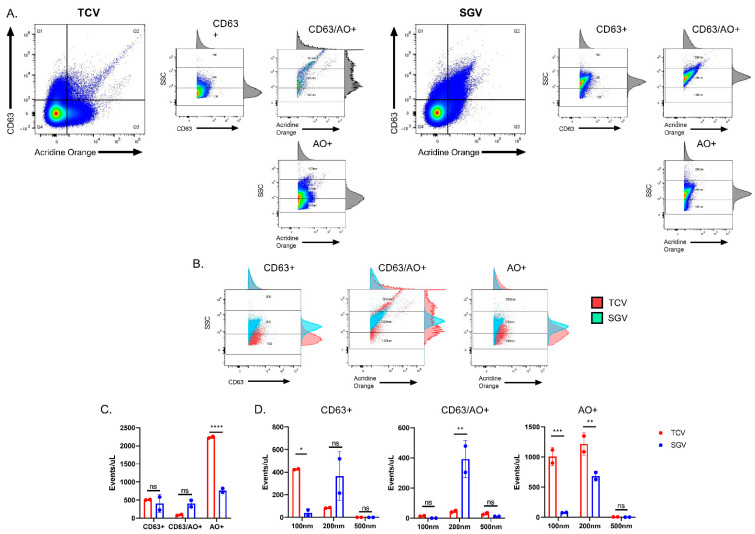
TCV and SGV differ in extracellular particle composition. (**A**) Flow virometry on equivalent titered SGV and TCV. Viral preparations were stained with anti-CD63 and AO and analyzed via flow cytometry. For the three positive quadrants (CD63+, CD63+/AO+, AO+) the positively stained events were assessed and shown for particle sizes. All events from the specified quadrants were analyzed by SSC vs. fluorescence. Size markers were established using fluorescent beads of the designated size. (**B**) Overlays of TCV and SGV positive quadrants. Fluorescence vs. SSC were plotted with gates representing 100, 200, and 500 nm sizes shown. (**C**) Quantification of events per microliter of equivalent titer and dilution TCV and SGV that were positive in each gate. (**D**) Size distribution of the differentially stained submicron particles. Graphical representation of the percent positive events in each quadrant shown in (**A**). Average +/− standard deviation of two replicates are shown and graphed based on the percentage of the sized particles within a quadrant. Representative plots shown. For (**B**), two-way ANOVA was performed with multiple comparisons. * *p* < 0.05, ** *p* < 0.01, *** *p* < 0.001, **** *p* < 0.0001, ns = not significant.

Nucleic acid positive events and size distributions differ between TCV and SGV (Figure 4) and uninfected cells or SG tissue (Figure 5 and Figure 6). To directly compare CD63+ and AO+ positive events between TCV and SGV stocks, equivalent titer virus stocks were stained as in Figure 5 and Figure 6 (Figure 7A,B). For TCV, CD63 and AO rarely co-stain, with most events being positive for either CD63 or AO (~500 and 2200 events/µL, respectively) and few double positives (~90 events/µL) (Figure 7A,C). This contrasts SGV where there was more equal distribution among positive events (~400 events/µL CD63+ and CD63+/AO+ and ~760 AO+) (Figure 7A,C). TCV and SGV have similar levels of CD63+ events, but TCV has considerably more AO+ than SGV (~2200 vs. 760) (*p* < 0.0001) (Figure 7A,B). In comparing the sizes of these different events, CD63+ events in TCV are predominantly below 200 nm in size (>80%), with CD63/AO+ double positives split more equitably among all three submicron sizes and AO+ single positive events almost evenly divided between 100 nm and 200 nm (49% vs. 50%) (Figure 7D). Because SGV has a highly homogenous particle size, CD63+ and CD63+/AO+ had >90% of events within the 200 nm size (Figure 7D). Only the AO+ events had <90% (88%) of events in 200 nm with ~12% in the smaller 100 nm gate (Figure 7D). These data point to the differences in particle sizes from SGV and TCV, with SGV generating a predominance of 200 nm particles and TCV often a balanced production of 100 nm and 200 nm particles.

**Figure 8:**
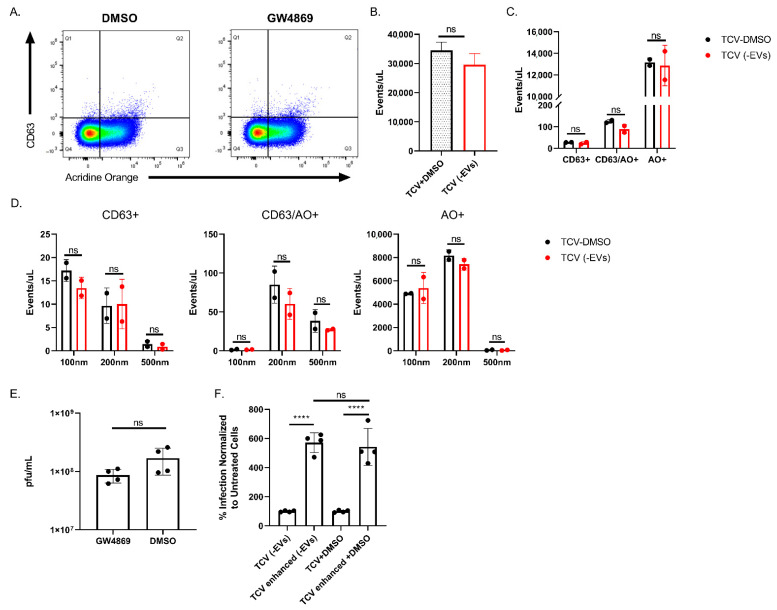
Exosome inhibition does not impact centrifugal enhancement. (**A**) Flow virometry comparison of CD63+ and AO+ events of virus produced from GW4869 and DMSO treated cells. Representative FACS plots are shown. (**B**) Event rate of TCV produced when treated with exosome inhibitor GW4869 (TCV (-EVs)) or DMSO control (TCV-DMSO). (**C**) Quantification of events per microliter that were positive in each gate of (**A**). (**D**) Particle size comparison of single and double positive CD63+ and AO+ events for DMSO treated TCV (TCV-DMSO) or EV-depleted TCV (TCV (-EVs)). (**E**) Viral titer of MCMV produced when incubated with EV-inhibitor (GW4869) or vehicle control (DMSO). (**F**) Centrifugal enhancement of viruses produced with or without EV-inhibitor. Virus was enhanced by centrifugation at 400× *g* and normalized to unenhanced, treated control. Representative plots shown. For all, n ≥ 2 and mean +/− standard deviation is shown. Student’s two-tailed *t*-test was performed for (**B**,**E**). For (**C**,**D**), two-way ANOVA was performed with multiple comparisons. One-way ANOVA with multiple comparisons was performed for (**F**). **** *p* < 0.0001, ns = not significant.

Due to the potential presence of EVs in isolated viral stocks, we sought to examine the impact of small EVs (exosomes) on centrifugal enhancement. Using the sphingomyelinase inhibitor, GW4869, we treated infected and uninfected cells to characterize the resulting viruses following exosomal diminution. The results of GW4869 treatment on submicron particles concentrated from uninfected flasks is shown in Supplementary Material Figure S1. Virus stocks treated with vehicle (DMSO) or exosome inhibitor (GW4869) were stained and analyzed via flow virometry (Figure 8A). Exosomal inhibition decreased total submicron particle event rate but failed to reach significance (Figure 8B). Further, GW4869 treatment failed to significantly alter submicron particle composition (Figure 8C) or the sizes of those particles (Figure 8D). Exosome inhibition was demonstrated on uninfected flasks in Supplementary Material Figure S1. Treatment did slightly decrease the amount of extracellular virus released by infected cells, but not significantly (*p* = 0.06) (Figure 8E). Also, despite inhibition of the exosome biogenesis pathway, the exosome-depleted virus still underwent enhancement to the same degree (~5-fold) as vehicle control and previous TCV stocks (Figure 1A and Figure 8F).

## 3. Discussion

Our results further contribute to our understanding of in vivo and in vitro MCMV, as well as the phenomenon of centrifugal enhancement. Speeds <800× *g* are still sufficient for enhancement to occur, and the centrifugal speed determines the degree of enhancement (Figure 1A). However, there was one point of difference versus previous observations. The initial description of enhancement of MCMV found that virus source (i.e., SGV and TCV) did not impact centrifugal enhancement [23]. In contrast, we found that in vivo derived virus was not enhanced to the same degree as in vitro derived virus (Figure 1C). This may be due to differences in the enhancement process as our centrifugation was performed at 400× *g* vs. their 2000× *g*. As our enhancement was performed at a slower speed, the fold change was significantly less (~50-fold vs. ~5-fold), and this may allow finer discrimination/differentiation of phenotypes between the two virus preparations. In support of our findings, the virulent strain of MCMV (K181), which was maintained by serial propagation through mouse salivary glands, was also reported to not enhance to the same degree as the cell-culture maintained Smith strain [28]. Several passages of K181 through cell culture were sufficient to reverse the phenotype and allow for centrifugal enhancement [28]. Our K181-derived viruses were differentially enhanced based upon passage in vivo or in vitro. This difference between virus sources was not due to a component within the salivary gland environment that directly inhibits enhancement as addition of uninfected salivary gland to TCV had no impact on unenhanced infection (i.e., control) or centrifugal enhancement groups (Figure 1D). We also found that the enhancement process occurs via the typical MCMV entry process as enhancement was impacted and unable to fully restore infection for sodium chlorate or heparin treated cells (Figure 2A,B). Enhancement was unaffected by the blockade of the endocytosis pathway (either chemically or by deletion of MCK2) (Figure 2C,D). This points to centrifugal enhancement utilizing the same entry process as typical infection (i.e., attachment to HSPGs), but with greater efficiency following a low-speed spin. This is in line with the increased adsorption mechanism hypothesized previously [23,24], whereby application of a centrifugal field during the infection process increases the retention of virus on the surface and leads to increased plaque formation. One explanation of why SGV does not enhance to the same degree as TCV, could be due to its lowered dependence on HSPGs for entry [15]. However, we also demonstrated that ex vivo generated BMDMV has a similar lowered dependence on HSPGs as SGV [15], yet its enhancement was similar to TCV. Failure of this correlation between HSPG usage and centrifugal enhancement, points to potentially another mechanism at play.

Since the discovery of centrifugal enhancement, cellular tropism and viral/host factors involved in entry into different cell types have been reported [51,53,57]. Tropism for MCMVs is determined based on the presence/absence of viral glycoproteins responsible for entry. Complexes containing either gO or MCK2 are responsible for entry by fusion or endocytosis, respectively [51,53]. These different entry processes allow for infection of fibroblasts via fusion and viral gO while viral MCK2 contributes to entry into epithelial/endothelial/monocytic cells via endocytosis [51,53]. The infected cell type likely determines the relative abundance/composition of these entry complexes [58]. SGV displays some restrictions in tropism when compared to the equivalent TCV [17]. If the lack of SGV enhancement is due to a restriction in tropism or alteration of tropism, we would expect a comparably tropism-restricted cell culture-derived virus to have lowered enhancement (Figure 1C). The less centrifugally enhanced SGV does not have tropism for macrophages/monocytes despite having an intact MCK2 [17]. Cell culture derived RM461 similarly does not have tropism for cell types requiring endocytosis, which includes monocytes/macrophages, and still shows enhancement on par with fully tropic TCV (Figure 2D) despite the loss of MCK2 [54,59]. RM461′s enhancement and SGV’s lack of enhancement eliminates tropism differences as the mechanism for centrifugal enhancement. Similarly, the HCMV strain AD169, which lacks a functional pentameric complex responsible for entry into non-fibroblast cell types, is still enhanced on human fibroblast cell lines [25]. Taken together, centrifugal enhancement is not due to changes in tropism of CMV, as viruses with full or restricted tropism are still capable of enhancement. This points to another component of the virus as being responsible for enhancement.

MCMV derived from cell culture and salivary glands are reported to differ in their composition of particles [19]. Infectious TCV consists of individual virions (~200 nm), non-infectious naked capsids (~200 nm), and infectious “multicapsid clusters” (≥500 nm) [19]. In contrast, the viral component of SGV is composed almost entirely of individual virions (~200 nm) [19]. We and others postulated that the larger clusters of viruses were responsible for the enhancement of TCV. Based on their size, they are more likely to be sedimented with slow speed centrifugation. Their entry into cells would also represent the delivery of multiple genomes in a single fusion event. However, when the multicapsid clusters are separated either by gradient [19] or filtration (Figure 3), enhancement still occurs. Because enhancement still occurred even when filtered through a 0.2 µm filter, particle sizes at 200 nm and below in TCV and SGV could be responsible for centrifugal enhancement.

The supernatant of infected (or uninfected cells) consists of a mixture of host-derived extracellular vesicles, many of which would easily pass through a 0.2 µm filter. EVs are known to deliver protein and RNA between cells, with DNA transfer more recently described [60,61]. Transfer of EVs between cells could serve to prime immune responses [62,63], alter host physiology [64,65,66], transfer infectious genomes [67,68], and reprogram host physiology to pro-viral states [47,69,70]. While still a burgeoning area of research, EVs from HCMV infected cells are known to package viral proteins intra-vesicularly and on their surface [44,45], and exosomes isolated from infected cells increase the rate of viral spread [37]. Up to now studies on the presence, composition, and impact of EVs from MCMV infected cells have been lacking. We found that MCMV alters the submicron composition of infected cells from tissue culture, increasing the number of submicron events (Figure 5B), nucleic acid positive events (Figure 5C,D), and globally decreasing CD63+ events (Figure 5C,D) for TCV vs. conditioned media. The increasing event rate in TCV is driven significantly by increased nucleic acid positive events (in 100 nm and 200 nm gates) (Figure 5C,D). For in vivo derived virus, there were no drastic differences in the types of particles secreted versus uninfected tissue homogenate, but infection globally increased the number of CD63+ and/or nucleic acid positive events (Figure 6A,C). This increase was only significant for nucleic acid positive events, but 200 nm events were significantly elevated in each stain (Figure 6C,D). With regard to TCV and SGV differences, nucleic acid single positive events in TCV greatly outnumber their SGV counterpart (Figure 7C). Within the CD63+ and AO+ portions, the TCV stock contains more <200 nm particles, but SGV generates few of these particles (<10%) (Figure 4 and Figure 7). This lack of smaller particles could be due to the rapid absorption of small EVs in vivo, low production in vivo, an increased production of naked capsids in cell culture, or a result of the salivary gland isolation process. CD63+ single-positive events likely represent EVs (not naked capsids or infectious virus). The relative abundance of TCV EVs below 200 nm in size may represent the “enhanceable element” not present in SGV stocks (Figure 7D). These EVs could increase the delivery of pro-viral products (i.e., host or viral protein, RNA, and/or DNA) to allow centrifugal enhancement. EVs utilize many of the same entry pathways as viruses, including fusion at cellular membranes and dependence upon HSPG-binding [38]. Centrifugation could be responsible for the increased retention of EVs on the cell surface much as was hypothesized for virual enhancement.

Pan or targeted inhibition of EVs is currently a technical challenge. Specific blockade of exosome release is possible and commonly performed using the drug GW4869 [37,46,71]. Exosomes have been implicated in the increased focal spread of HCMV, post-initial infection [37]. We provide evidence that an EV inhibitor (GW4869) does not significantly impact the generation of cell culture virus stocks (Figure 8A–D). Virus produced with and without inhibitor is dominated by nucleic acid positive events (Figure 8A,C). EV inhibition was performed at similar concentrations and timepoints as previously reported, and differences were observed in submicron particles of uninfected cells following GW4869 treatment (Supplementary Material Figure S1). We found a slight but non-significant decrease in viral titer (Figure 8E), again matching a previous report [37]. Finally, EV-depletion failed to prevent centrifugal enhancement (Figure 8F). Based on these results, we would discount the involvement of exosomes in the process of centrifugal enhancement. Unfortunately, we cannot rule out the contribution of other, larger EV types. Similarly, while CD63 is a prominent marker of EVs, understanding of the full EV complexity has yet to be resolved, and the contribution of other EV types may be significant.

We present further evidence for the distinction of in vivo and in vitro MCMVs. MCMV derived from infected salivary glands undergoes enhancement to a lesser degree than cell culture viruses. While this could be driven by differences in HSPG interactions, MCMVs resistant to HSPG-binding inhibitors still displayed enhancement [15]. Consequently, we sought to determine the differences in EVs and submicron composition of virus preparations and their impact on centrifugal enhancement. Following MCMV infection, the secretion of extracellular vesicles and submicron particles by host cells are altered. Despite these alterations and the presence of CD63+ submicron particles, exosomal inhibition had no noticeable impact on centrifugal enhancement. GW4869 treatment did slightly (but not significantly) lower the production of submicron particles and extracellular viral titer of infected cells. Thus, despite likely containing EVs of varying origins with potential pro-and anti-viral roles, the exosomes contained within MCMV viral stocks are not responsible for spin enhancement of CMV. Although we were not able to identify the factor responsible for enhancement, we were able to demonstrate further differences between MCMVs derived in vivo and in vitro. The host or viral factors contained within EVs represent an important puzzle piece for further dissecting differences between TCV and SGV viruses. Although EVs may not play a role in centrifugal enhancement, we have shown differences in their generation between in vivo vs. in vitro grown MCMVs. The translatability of tissue culture EVs to in vivo disease is an important question going forward, and one that the murine model of CMV is uniquely suited to address.

## 4. Methods

### 4.1. Cells and Viruses

Low passage (<20) MEF 10.1 cells [72] were used to generate and titer viral stocks. Cells were maintained in Dulbecco’s modified Eagle medium (DMEM) with 10% triple 0.1 µm filtered Fetalclone III serum (Cytiva, Marlborough, MA, USA), and 1% penicillin/streptomycin and l-glutamine, each. Viral strains RM 4503, RM 461, and K181 were used for infections and plaque assays [54,73]. Once 100% cytopathic effect was observed, viral supernatants were clarified by 2× 400× *g* spins at 4 °C for 20 min. Virus was then pelleted by centrifugation at 20,000× *g* for 2 h at 4 °C. Viral pellets were resuspended in cell culture media and sonicated 2× at 20% amplitude for 30 s, aliquoted, and stored at −80 °C until use. Generation of both salivary gland and bone marrow-derived macrophage virus stocks has been previously reported [15]. Briefly, salivary gland (SG) virus stocks were first homogenized in a dounce homogenizer before being centrifuged and sonicated as above. Bone marrow-derived macrophages (BMDM) were generated by plating bone marrow collected from mouse femurs in RPMI 1640 in non-treated sterile dishes. Non-adherent cells were removed 3 h later by aspiration. Cells were stimulated for 7 days in the presence of 10 ng/mL of M-CSF (Peprotech, Cranbury, NJ, USA). ~3.5 days post-isolation cells were washed 2× with cold PBS and replaced with fresh media supplemented with M-CSF. Cells were >95% pure as assessed by flow cytometry. BMDMs were infected with virus and once 100% CPE reached (~14 days post infection (dpi)), virus was isolated as described above for tissue culture virus. “Concentrated media” or “conditioned media” was obtained by centrifuging fresh cell culture media or spent media as above for tissue culture virus.

### 4.2. Animals

BALB/cJ mice were obtained from Jackson Laboratory (Bar Harbor, ME, USA) and housed in specific-pathogen free environment at the University of Tennessee. For SG-derived viruses, mice were infected with ~1 × 10^6^ PFU intraperitoneally (i.p.) and salivary glands harvested 14 days post-infection. All procedures were reviewed and approved by the Institutional Animal Care and Use Committee at the University of Tennessee.

### 4.3. Plaque Assays and Centrifugal Enhancement

Plaque assays were performed in 12-well tissue culture treated plates. MEF 10.1 cells were seeded at ~1 × 10^5^ cells per well and allowed to reach confluency overnight. Spent media was aspirated and viral inoculum was added to “enhanced” wells, and plates were centrifuged at 400–800× *g* for 10 min at 4 °C. Virus was added to control/non-enhanced wells and plates placed in 37 °C CO incubator for 1 h. After 1 h, viral inoculums were removed and replaced with a carboxymethylcellulose (CMC) overlay. Plates were incubated for a further 5 days, fixed and stained with Coomassie blue, and plaques counted. Slight modifications were made for experiments involving entry inhibitors (i.e., sodium chlorate, ammonium chloride, and heparin sodium salts). Sodium chlorate was added at 50 mM, and incubated with cells overnight at 37 °C. Ammonium chloride was added at 50 mM, and incubated with cells for 2 h. Heparin sodium salt was added at 50 µg/mL and incubated with cells for 30 min at 4 °C.

### 4.4. Flow Virometry

Virus preparations were diluted 1:10 in 0.2 µm filtered ddH_2_O. Extracellular DNA and RNA was removed by addition of DNase/RNase (DNase I, ~10 U/mL) (MilliporeSigma, Burlington, MA, USA) (RNase A, 100 µg/mL) (Thermo Fisher Scientific, Waltham, MA, USA) and incubated at 37 °C for 30 min. Following DNase/RNase treatment, virus was fixed by mixing 1:1 with BioLegend fixation buffer (BioLegend, San Diego, CA, USA) (4% paraformaldehyde) and incubating at 4 °C for 10 min. After fixation, virus was stained with anti-EV antibodies (anti-CD63, PE-Cy7, NVG-2 clone) (BioLegend, San Diego, CA, USA) and/or nucleic acid stain acridine orange (1 µg/mL). Stained and unstained virus was diluted 1:10 and serially diluted 1:2 generating 5–6 dilutions for flow cytometric analysis. In addition to unstained controls, antibody and dye only controls were generated at equivalent dilutions to samples to determine potential for dye/antibody aggregates and false positives. To analyze samples, a Cytek Northern Lights flow cytometer (Cytek Biosciences, Fremont, CA, USA) equipped with 488 and 405 nm lasers was used. Violet SSC was used to trigger the threshold. Sub-micron particle size reference beads were used to establish approximate size gates (Thermo Fisher Scientific, Waltham, MA, USA) with SSC vs. fluorescence. Sample event rate vs. dilution was used to determine linearity and minimize the possibility of “swarms”. Additional 1:2 dilutions were performed as necessary to reach event rate linearity. All flow analysis was performed in FlowJo ver. 10.8 (FlowJo, Becton, Dickinson and Company, Ashland, OR, USA).

### 4.5. Exosome Inhibition

Exosome inhibition was performed as in [37]. MEF 10.1 cells were grown to confluency in a T-175 flask. Two days before infection, cells were pre-treated with either GW4869 (8 µm) (Tocris Biosciences, Bristol, UK) or DMSO (dimethylsulfoxide, vehicle control). Prior to infection, media was replaced, and cells were infected at ~0.1 MOI. Three hours after infection, GW4869 or DMSO was added back to the flasks at 2.5 µm. GW4869 was replenished (2.5 µm) every other day until 100% CPE was reached (6–7 dpi), at which point virus was harvested as above.

### 4.6. Statistics

Each experiment consisted of two or more independent experiments with 3 replicates per group, unless indicated otherwise. Individual data points are shown for all graphs, with mean and standard deviation error bars. Statistical significance was determined by one or two-way ANOVA with multiple comparisons or one-tailed Student’s *t*-test where appropriate. All graphs and statistical analyses were performed in GraphPad Prism ver. 9 (GraphPad Software, La Jolla, CA, USA). Significance: * = *p* < 0.05, ** = *p* < 0.01, *** = *p* < 0.001, **** = *p* < 0.0001, ns = not significant.

## Data Availability

All relevant data is available upon request.

## Supplementary Materials

The following are available online at https://www.mdpi.com/article/10.3390/pathogens10121577/s1, Figure S1: Exosome inhibition alters EVs produced by uninfected MEF 10.1 cells.
