# Supplementary material for: MCMV Centrifugal Enhancement: A New Spin on an Old Topic
Source: Pathogens. 2021 Dec 3;10(12):1577. doi: 10.3390/pathogens10121577 (PMC8705575; doi:10.3390/pathogens10121577)
Supplement: Supplementary file 1 [file pathogens-10-01577-s001.zip › pathogens-1425131-supplementary.pdf]

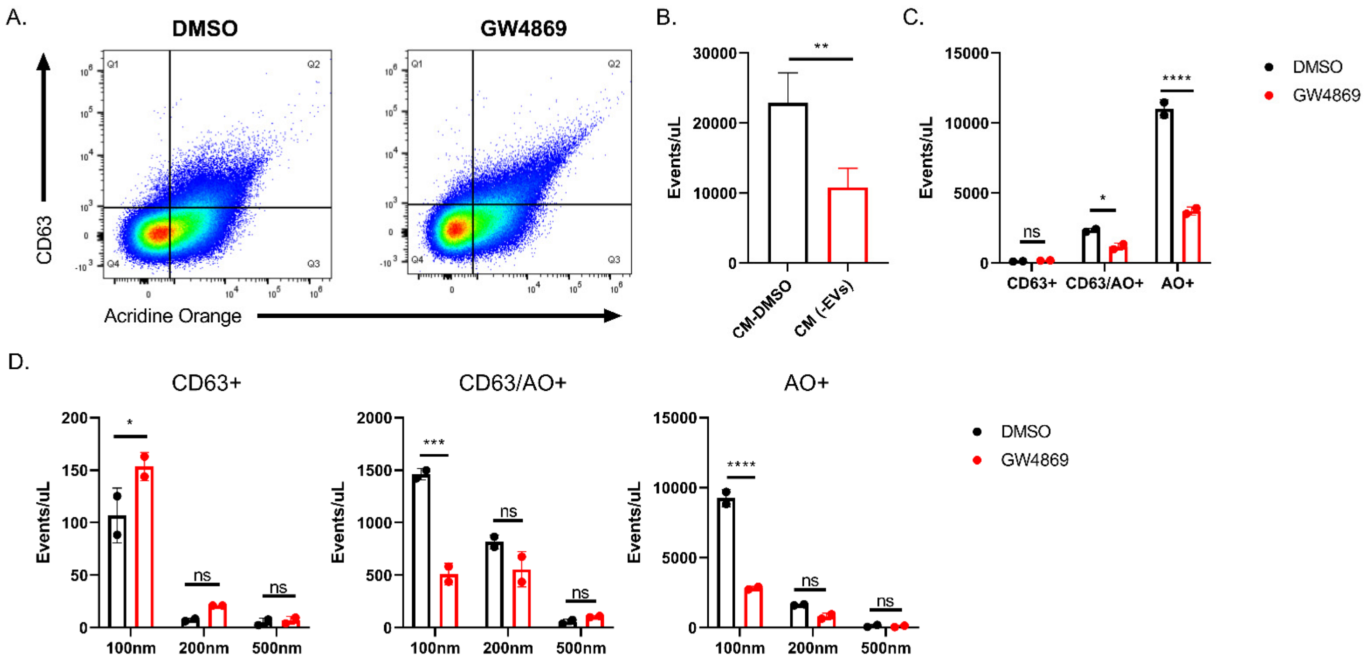

Figure S1. Exosome inhibition alters EVs produced by uninfected MEF 10.1 cells. (**A**) Comparison of CD63+ and AO+ events for conditioned media treated with EV inhibitor (GW4869) or vehicle control (DMSO). Representative FACS plots are shown. (**B**) Event rate of similarly produced conditioned media depleted of EVs (CM (-EVs)) or control (CM-DMSO). (**C**) Quantification of events per microliter that were positive in each gate of (**A**). (**D**) Particle size comparison of single and double positive CD63+ and AO+ events for treated and untreated conditioned media. Representative plots shown. For all, n ≥ 2 and mean +/− standard deviation shown. Student's two-tailed *t*-test was performed for (**B**). For (**C**,**D**), two-way ANOVA was performed with multiple comparisons. * $p < 0.05$, ** $p < 0.01$, *** $p < 0.001$, **** $p < 0.0001$, ns = not significant.
